# Adult Female With Abdominal Distention

**DOI:** 10.7759/cureus.13829

**Published:** 2021-03-11

**Authors:** Sarah E Frasure, Joel J Lange, Marya AlSamman, Ali Pourmand

**Affiliations:** 1 Department of Emergency Medicine, George Washington University School of Medicine and Health Sciences, District of Columbia, USA

**Keywords:** gastric outlet obstruction, abdominal ultrasound, gastric distention

## Abstract

Gastric outlet obstruction (GOO) is a rare diagnosis that can be challenging to make as its symptoms, which include abdominal distention, nausea, and persistent vomiting, often overlap with many other acute abdominal pathologies. Point-of-care ultrasound (POCUS) can help the clinician identify gastric outlet obstruction in patients who present to the emergency department (ED). Sonographic identifiers include a markedly dilated stomach that is filled with both hyper- and hypoechoic contents and may extend into the lower abdomen in the pelvic views.

## Introduction

Gastric outlet obstruction (GOO) is a rare clinical syndrome that typically presents with epigastric abdominal pain, abdominal distention, and post-prandial vomiting due to a mechanical obstruction of the gastric outlet. Obstruction of gastric outflow has several etiologies including peptic ulcer disease (PUD), malignancy, pancreatitis, hypertrophic pyloric stenosis, and foreign bodies, such as a bezoar or large gallstone (Bouveret syndrome) [1-3]. Historically, PUD accounted for up to 90% of cases of gastric outlet obstruction [1-2]. With advancement in the medical treatment of PUD resulting in the declining number of such cases, however, malignancy has become the more common cause of GOO [3-5]. Of the malignant causes of GOO, pancreatic adenocarcinoma, with extension into the duodenum or stomach, and distal gastric cancer, are the most common [6-9]. In this case report, we discuss the use of point-of-care ultrasound (POCUS) in the evaluation of a patient presenting to the emergency department (ED) with abdominal pain, hypotension, and tachycardia, who was diagnosed with GOO.

## Case presentation

**Figure 1:**
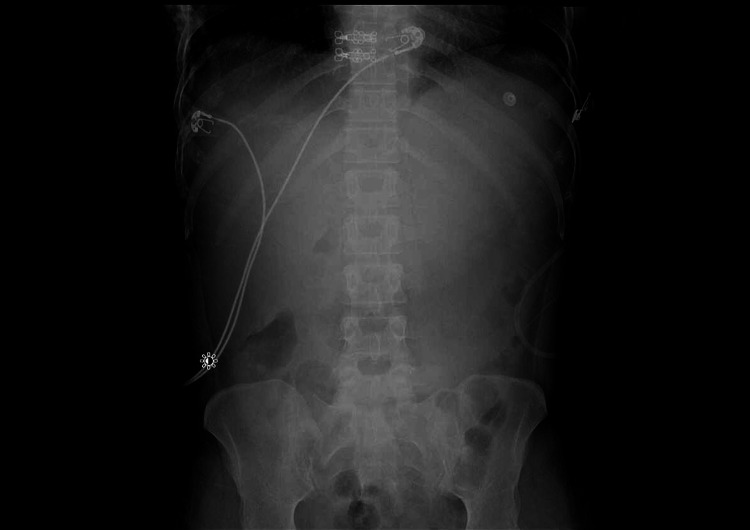
The bowel gas pattern appears normal. No free intra-peritoneal air is present.

A 33-year-old female with multiple prior hospitalizations for heavy alcohol use presented to the ED with diffuse abdominal pain, nausea, and vomiting. Symptoms had been ongoing for three days. Vitals signs were notable for a heart rate of 150 beats per minute, a blood pressure of 90/54 mmHg, and an oxygen saturation of 99% on room air. She was afebrile. On physical examination the clinician noted that the patient was in severe distress with dry mucous membranes and a markedly distended abdomen that was exquisitely tender to palpation diffusely. The patient was administered intravenous fluids, opioid pain medication and antiemetics. Bloodwork was obtained and an abdominal x-ray was initially performed (Figure 1), which demonstrated no evidence of free air under the diaphragm and a normal gas pattern.

**Figure 2:**
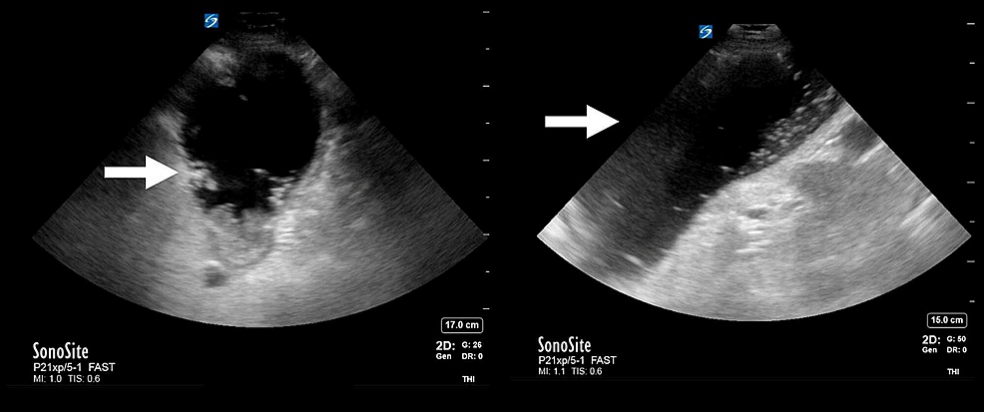
Enlarged stomach (see arrows) with stomach contents in left upper quadrant and left lower quadrant of the abdomen.

Given her physical exam findings of abdominal distention and diffuse tenderness to palpation, however, the physician performed an abdominal ultrasound with a Sonosite X-Porte (Sonosite Inc., Bothell, WA, USA). The phased array transducer (P21xp; 5-1MHz) with the ‘abdominal’ preset was chosen for the exam. The patient was placed in the supine position and the clinician performed a Focused Assessment with Sonography for Trauma (FAST) examination which was immediately notable for a very large stomach that was initially identified in the left upper quadrant (LUQ) next to a normal-appearing spleen and left kidney. Furthermore, the stomach extended into the left lower quadrant (LLQ) and pelvis (Figure 2). 

**Figure 3:**
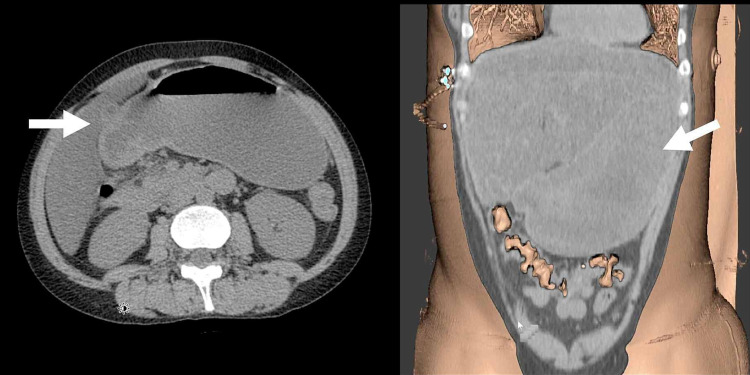
Sagittal and coronal CT imaging demonstrates gastric outlet obstruction with dilatation of the stomach and distal esophagus (see arrows).

The remainder of the FAST examination was unremarkable. Given the highly abnormal appearance of the stomach, concern for gastric outlet obstruction was raised, and the patient underwent computed tomography (CT) imaging which confirmed the diagnosis of GOO with dilation of the stomach and distal esophagus (Figure 3).

**Figure 4:**
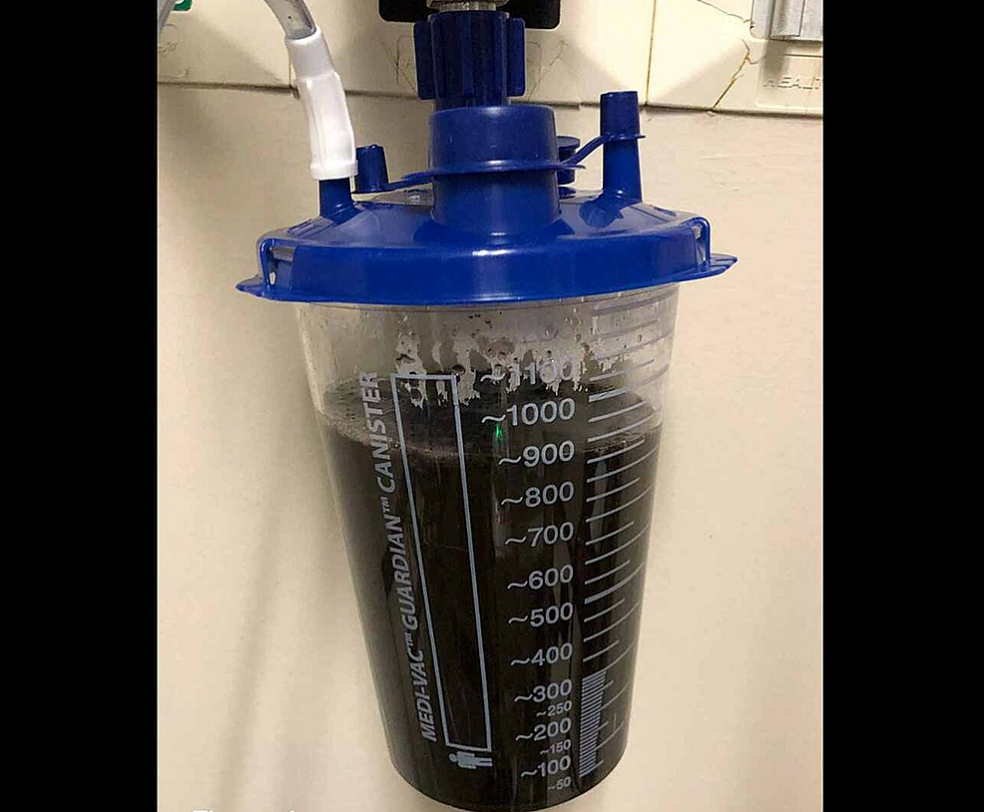
Nasogastric tube output in the ED

A nasogastric tube (NGT) was subsequently placed by the provider with immediate output of 900cc of gastric content (Figure 4).

Bloodwork was notable for an anion gap > 39 and a lipase value > 9700. Her bicarbonate level was < 5 and a urine pregnancy test was negative. Both the surgical and gastroenterology services were consulted by the clinician; the patient was ultimately admitted to the intensive care unit in critical condition with a diagnosis of gastric outlet obstruction secondary to acute pancreatitis with severe metabolic acidosis thought to be the result of chronic heavy alcohol use.

## Discussion

Point-of-care ultrasound has enjoyed increasing utility in the emergency department in the last decade and has become a crucial tool in the evaluation of patients, especially those who are critically ill. POCUS may provide a rapid and noninvasive method to diagnose possible gastric outlet obstruction [10]. A case report published in 2017 highlighted the use of POCUS to diagnose GOO. The report describes the use of a curvilinear probe placed in the epigastric area to identify a dilated, fluid-filled structure [10]. It also highlights the importance of using ultrasound to differentiate between GOO and a small bowel obstruction (SBO). Similarly, a case series of three patients discussed the use of POCUS in making the diagnosis of GOO. In all three patients, the stomach had a distinct appearance on POCUS resembling a “black and white cookie” [9]. The appearance seemed to be caused by a hyperechoic meniscal layer of gastric contents that contained an internal division of anechoic as well as hyperechoic areas [9]. In both studies, a significantly distended stomach was noted on the ultrasound.

As mentioned in both of the previous case reports, however, utilizing POCUS in the diagnosis of GOO does have limitations. A dilated stomach cavity can be found in both bowel obstructions and GOO and it is crucial to examine the entire abdominal area while using POCUS to evaluate for GOO [9-10]. When there is no evidence of a small bowel obstruction, a presumptive diagnosis of GOO can be made and further workup should be pursued with CT imaging and surgical or gastroenterology consultation. It is important to note that the characteristic rounded borders of the free fluid in our ultrasound images point to an intra-luminal location (stomach, bowel, cyst) rather than the existence of intra-peritoneal free fluid. The characteristic location of this intra-luminal fluid in the LUQ just anterior to the spleen and left kidney also helps the clinician to identify the fluid as gastric in origin. Finally, POCUS is valuable in serial examinations to monitor the resolution of the obstruction depending on the etiology or after nasogastric tube placement [10]. 

Certainly, GOO can be a challenging diagnosis to make given the non-specific nature of the symptoms which include nausea, vomiting, and abdominal distention, all of which are present in many other abdominal pathologies. Abdominal CT imaging is the standard diagnostic tool for identifying GOO in the emergency department and can help to identify the underlying etiology. Findings on abdominal CT scan include gastric distention with retained material in the gastric lumen and an air fluid level [11]. Although CT imaging may be more sensitive than POCUS, it could result in a delay in diagnosis and increased morbidity. POCUS appears to be a promising alternative for the rapid assessment of a patient with a possible GOO. Earlier diagnosis can lead to more specific patient evaluation, specialty consultation, and medical treatment. However, it is critical that physicians are properly trained in POCUS and aware of the limitations of this imaging modality [12].

## Conclusions

Gastric outlet obstruction is a rare diagnosis that can be challenging to make as its symptoms, which include abdominal distention, nausea, and persistent vomiting, often overlap with many other acute abdominal pathologies. Point-of-care ultrasound, however, can help the clinician identify GOO in patients who present to the ED. Sonographic identifiers include a markedly dilated stomach that is filled with both hyper- and hypoechoic contents and may extend into the lower abdomen in the pelvic views.

## References

[1] Khullar SK, DiSario JA (1996). Gastric outlet obstruction. Gastrointest Endosc Clin N Am.

[2] Appasani S, Kochhar S, Nagi B, Gupta V, Kochhar R (2011). Benign gastric outlet obstruction-spectrum and management. Trop Gastroenterol.

[3] Johnson CD (1995). Gastric outlet obstruction malignant until proved otherwise. Am J Gastroenterol.

[4] Shone DN, Nikoomanesh P, Smith-Meek MM, Bender JS (1995). Malignancy is the most common cause of gastric outlet obstruction in the era of H2 blockers. Am J Gastroenterol.

[5] Johnson CD, Ellis H (1990). Gastric outlet obstruction now predicts malignancy. Br J Surg.

[6] Tendler DA (2002). Malignant gastric outlet obstruction: bridging another divide. Am J Gastroenterol.

[7] Samad A, Khanzada TW, Shoukat I (2007). Gastric outlet obstruction: change in etiology. Pak J Surg.

[8] Chowdhury A, Dhali GK, Banerjee PK (1996). Etiology of gastric outlet obstruction. Am J Gastroenterol.

[9] Cohen A, Foster M, Stankard B, Owusu M, Nelson M (2018). The "black-and-white cookie" sign - a case series of a novel ultrasonographic sign in gastric outlet obstruction. Clin Pract Cases Emerg Med.

[10] Gottlieb M, Nakitende D (2017). Identification of gastric outlet obstruction using point-of-care ultrasound. Am J Emerg Med.

[11] Awan A, Johnston DE, Jamal MM (1998). Gastric outlet obstruction with benign endoscopic biopsy should be further explored for malignancy. Gastrointest Endosc.

[12] Blanco P, Volpicelli G (2016). Common pitfalls in point-of-care ultrasound: a practical guide for emergency and critical care physicians. Crit Ultrasound J.

